# Growth rate assays reveal fitness consequences of β-lactamases

**DOI:** 10.1371/journal.pone.0228240

**Published:** 2020-01-31

**Authors:** Fabian Santiago, Evin Doscher, Jay Kim, Manel Camps, Juan Meza, Suzanne Sindi, Miriam Barlow

**Affiliations:** 1 Department of Applied Mathematics, University of California Merced, Merced, CA, United States of America; 2 Department of Molecular and Cellular Biology, University of California Merced, Merced, CA, United States of America; 3 Department of Microbiology and Environmental Toxicology, University of California at Santa Cruz, Santa Cruz, CA, United States of America; 4 Department of Biomolecular Engineering, University of California at Santa Cruz, Santa Cruz, CA, United States of America; Rabin Medical Center, Beilinson Hospital, ISRAEL

## Abstract

Antibiotic resistance is a powerful model for studying evolutionary biology and population genetics. For the purpose of these evolutionary studies, fitness data have been approximated through susceptibility testing methods designed for clinical use in providing appropriate antibiotic therapies. An alternative approach for measuring fitness of microbes has experienced growing popularity: growth rates are a highly sensitive approach for measuring the fitness effects of antibiotics and resistance genes, but they differ from susceptibility testing in that a single concentration of antibiotic is used for the assay. Here we show that despite this key difference, the results of growth rates correlate well with clinical determination of resistance by minimum inhibitory concentration (MIC), while providing the sensitivity required for direct input as fitness values into mathematical models. This means that growth rates at a single sublethal inhibitory concentration can help us understand the fitness effects that result in clinical antibiotic resistance. By measuring the growth rates of sequenced clinical isolates obtained from Dignity Health Mercy Medical Center, we detected the fitness effects of individual resistance genes on bacteria exposed to different antibiotics. In our study, the CTX-M-15 gene conferred the highest fitness in assays with cephalosporins. These results show the strong fitness benefit conferred by CTX-M-15.

**Non-technical summary:**

Antibiotic resistance is a global human health problem. We partnered with Dignity Health Mercy Medical Center to study antibiotic resistance in clinical isolates. We tested whether growth rates, a sensitive assay used to measure the fitness of bacterial samples, correlate with a clinical test to measure antibiotic resistance. We found a strong correlation between these two methods suggesting that growth rates could be reliably applied to evolutionary studies of clinically relevant problems. Moreover, the sensitivity of the growth rates assay enabled us to identify fitness effects of specific antibiotic resistance genes.

## Introduction

Antibiotic resistance has become a powerful model system for studying evolutionary biology. The emergence, mutation and selection of antibiotic resistance genes has created a nearly unique opportunity to study fitness [1], adaptation [2],[3], pleiotropy [4], epistasis [5], adaptive landscapes [6], and evolutionary potential [7]. Studying these specific aspects of antibiotic resistance requires the measurement of fitness of bacterial isolates. Although antibiotic clinical classifications were not designed to measure fitness, antibiotic susceptibility testing has been heavily used as an approximation of fitness [1]. The justification in the field is that as resistance to an antibiotic increases, the fitness of organisms exposed to that antibiotic is likewise increasing. More recently, growth rates [8] have been implemented as a direct measurement of fitness [9] but without direct experimental connection to susceptibility testing results. While this method has been rapidly catching on, and its results are being used to answer important questions about evolution [10], there are few studies investigating the effects of this change in methodology upon the data and results to which we now have access. In this study, we investigate the correlation of growth rate assays to susceptibility testing and find that growth rates are a much more sensitive method for assessing fitness in bacteria.

Clinical testing of antibiotic resistance is critical for the development of effective treatment options. By determining the susceptibility of an isolate to an antibiotic, health care providers can properly administer antibiotics [11]. Three common methods used to detect resistance among bacterial isolates are: disc diffusion, E-testing, and broth dilution minimum inhibitory concentrations (MICs) [12]. In a clinical setting, bacterial isolates are classified as resistant, intermediate, or susceptible (RIS) to individual antibiotics. While these methods are useful for detecting antibiotic resistance, they lack the sensitivity for precise comparison of fitness in different antibiotics. Moreover, these clinical classifications are not amenable to quantitative analyses and were not designed to measure fitness.

A more sensitive measurement that reflects fitness is bacterial growth rate, which is a measure of the rate at which bacteria go through binary fission. Bacterial growth is characterized by four phases: lag phase, exponential phase, stationary phase, and death phase [13]. Typically, bacterial growth rates measure the exponential phase since this is the period in which the most growth occurs in the bacterial population. Growth rates have not yet been compared to clinical assays and it is unknown how growth rates correlate with clinical susceptibility assays. One of the goals of this study is to investigate if growth rates correlate with clinical resistance classification.

We hypothesized that growth rates can provide evidence about the relative contributions of resistance genes to the susceptibility of clinical isolates [9]. We reasoned that a low bacterial growth rate in the presence of an antibiotic would indicate susceptibility to that antibiotic, whereas a high bacterial growth rate would indicate elevated resistance to that antibiotic [9]. In either case, growth rates are a more quantitative measure of susceptibility to antibiotics and are more amenable to mathematical modeling and analysis. We additionally hypothesized that the quantitative sensitivity of growth rates would make it possible to generate reliable estimates about the predicted effect that the presence of resistance genes has on fitness.

To investigate these relationships, we partnered with Dignity Health Mercy Medical Center. Since 2013 we have collected patient isolates consisting primarily of *E*. *coli* from urinary tract infections (UTIs) that are resistant to extended spectrum beta-lactamases (ESBLs). While antibiotics are usually an effective treatment against UTIs, the rise of antibiotic resistant bacteria is starting to limit their effectiveness. Moreover, many isolates of bacteria are resistant to multiple antibiotics so identifying appropriate treatments remains a challenge [14]. We have previously used these patient isolates to study broad trends in resistance [15]; in this work we study the correlation between isolate growth rates and clinical resistance classifications as well as the presence of known resistance genes.

### β-lactamases and antibiotic resistance

β-lactamase genes are among the most prevalent resistance genes in UTIs. This study considers the effects of three β-lactamases: TEM-1, CTX-M-15, and OXA-1. We consider these three specifically because they are the most common resistance genes in our isolates (Table 5, Fig 2, and accompanying text). Although the resistance gene *ampC* occurred with high frequency in our isolates we did not include it in our analysis because it is almost always found within the chromosome of *E*. *coli* and usually not expressed.

Historically the most common β-lactamase has been TEM, which has accounted for approximately 90% of ampicillin resistance in *E*. *coli* [16]. TEM-1, the most common TEM causes penicillin resistance, while most of the 200+ variants that have evolved from it confer cephalosporin resistance as well. Based on structure, TEMs have been categorized as Class A β-lactamases. Conformational changes in the active site of the enzyme, caused by amino acid substitutions result in the different resistance phenotypes (cephalosporin resistance) conferred by extended spectrum β-lactamases (ESBLs). More recently, another family of Class A resistance genes, the CTX-Ms, has been replacing TEM β-lactamases in clinical isolates [16], [17], [18]. These confer cephalosporin resistance, including cefepime resistance at levels equal to or surpassing TEMs. This trend has caused a reduction of available treatment options and primary treatment has shifted to carbapenems. We note, TEM and CTX-M are structurally similar as they are both Class A β-lactamases [19].

OXA β-lactamases are Class D serine β-lactamases that were named for their ability to hydrolyze oxacillin [20]. They are typically located on large plasmids and have been present in this location before the antibiotic era [21]. While they are not the most efficient at cephalosporin hydrolysis, these resistance genes eventually evolved the ability to confer resistance to cephalosporins and carbapenems [20].

## Methods

### Ethics statement

This project was evaluated by the IRB committees of both UC Merced and Dignity Health Mercy Medical Center and was classified as an exempt study by both. The isolates were isolated in the course of normal patient care, and provided to us after their clinical usefulness was fulfilled. Furthermore, the samples were de-identified, and for these reasons patient consent was not required.

### Hospital isolates

The isolates used in this study were collected from patients admitted to Dignity Health Mercy Medical Center in Merced, California. They are representatives of the different resistance phenotypes and species that were collected from the hospital. Our goal in selecting these were to make sure each phenotype was represented at least once. For each sample we recorded: the date of sample isolation, the age and gender of the patient, the species of the bacteria, the source of the isolate, and its phenotype: resistant, intermediate, or susceptible for 16 different antibiotics (ampicillin, ampicillin/sulbactam, piperacillin/tazobactam, cefazolin, ceftazidime, ceftriaxone, cefepime, ertapenem, imipenem, amikacin, gentamicin, tobramycin, ciprofloxacin, levofloxacin, nitrofurantoin and sulfamethoxazole/trimethoprim) using standard breakpoints CLSI M100-S26 (2015).

### Growth rate assays

Growth rate inocula were taken from standing overnight cultures and diluted to a final working concentration of 10^5^ cells per mL in Mueller Hinton (MH) broth. The growth rate assay was performed in a BIOTEK (Model# 267638) spectrophotometer for 22 hours on stationary cultures at a temperature of 37° C at a wavelength of 600nm. Reads were collected every 20 minutes and after the 22-hour incubation period the optical density (OD) readings were then converted into growth rates using the freely available GrowthRates software package [8]. The growth rate for each isolate was measured with six replicates for each antibiotic and control (no antibiotic). The cephalosporin antibiotics used were ceftazidime (CAZ), ceftriaxone (CRO), cefepime (FEP), all at a concentration of 64 μg/mL. Isolates were also tested against ampicillin (AMP) at 32 μg/mL. We chose these concentrations because after measuring the growth rates of 213 separate clinical isolates, we found that these concentrations provided a broad range of growth rates that indicated phenotypic differences between isolates (S1–S3 Figs). It was necessary to use concentrations that in some cases exceeded CLSI breakpoints for resistance because the variance in growth rates was insufficient at other concentrations to detect fitness differences.

### Statistical analysis of growth rates

We analyzed isolate growth rates in the presence of an antibiotic as a function of antibiotic susceptibility (S/R) and genetic differences (presence or absence of resistance alleles). We performed all analyses with R version 3.3.2 [22] and used α = 0.01. In order to control for multiple statistical tests, we used The False Discovery Rate (FDR) Controlling Procedure [23], which is a Bonferroni-type multiple testing procedure for both Welch’s t-tests and the Shapiro-Wilk tests. Applying this procedure, with a false discovery control level of q* = 0.05, we report as significant only those results that remained significant after using FDR.

**Fig 1:**
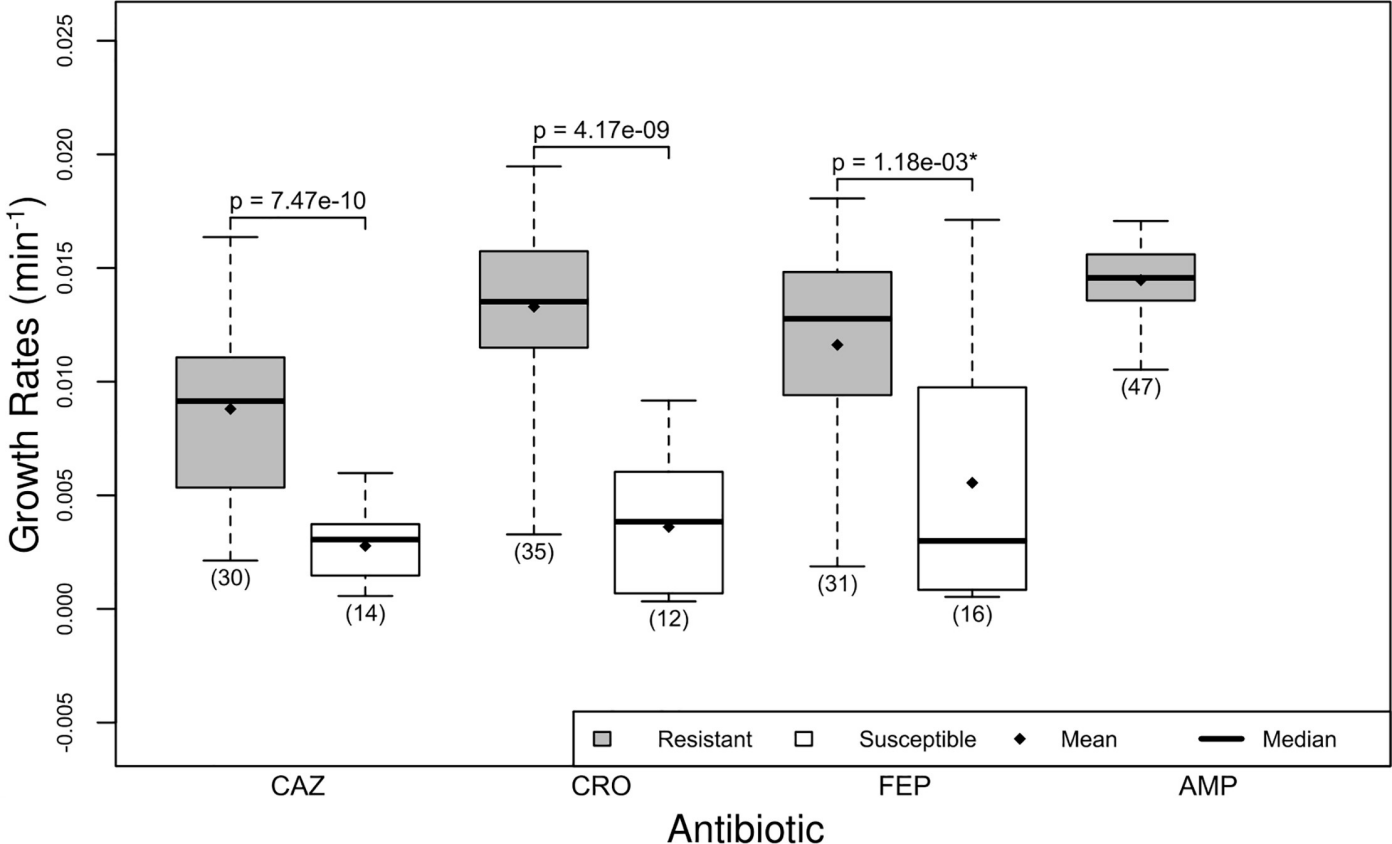
Resistant isolates grow faster than susceptible isolates. There are two boxplots per antibiotic, the first (gray) shows the distribution of growth rates for isolates that are resistant to the antibiotic, and the second (white) shows the distribution of growth rates for isolates that are susceptible to the antibiotic. The boundaries on the boxes indicate the 25^th^ (Q1) and the 75^th^ (Q3) percentiles, the line in the box represents the median, the diamond represents the arithmetic mean, and the whiskers indicate the minimum (below) and maximum (above) growth rate. The number of isolates used to create each boxplot is given in parenthesis. The asterisk indicates that one (or both) of the groups of growth rates under consideration did not pass a test for normality (Table 1).

We tested the growth rates in each condition using the Shapiro-Wilk test, which rejects the null hypothesis of normality based on the skew and kurtosis of the sample [24]. Most of our data did not deviate from normality (Tables 1 and 2), which allowed us to compare the means between conditions using Welch’s t-test [25]. The skew we observed in growth rates for cefepime (Fig 1), may be due to greater responsiveness to slight concentration differences in cefepime than in other antibiotics. Though the growth rates data may have different variances, equal variance is not a requirement of Welch’s t-test.

**Table 1:** P-values for Shapiro-Wilk normality test in each condition. For the row labeled “Sensitivity”, the first column corresponds to resistance to the antibiotic (R), and the second column corresponds to susceptibility to the antibiotic (S). For the rows labeled with a resistance gene (CTX-M-15, TEM-1, OXA-1) the first column indicates the presence of a resistance gene (+), while the second column indicates the gene is not present (-). All cases except growth rates in the presence of cefepime in the second column (-/S) indicate normality (with p < 0.01).

	R/ (+)	S/ (-)
	Ceftazidime	
Sensitivity	0.67300	0.4678
CTX-M-15	0.73220	0.6183
TEM-1	0.10680	0.1293
OXA-1	0.61540	0.1417
	Ceftriaxone	
Sensitivity	0.32270	0.1184
CTX-M-15	0.04209	0.1165
TEM-1	0.27030	0.0792
OXA-1	0.24300	0.0979
	Cefepime	
Sensitivity	0.02671	2.925e-3
CTX-M-15	0.03977	4.293e-4
TEM-1	0.01576	7.000e-3
OXA-1	0.08910	3.577e-3

**Table 2:** P-values for Shapiro-Wilk normality test with combined resistance genes. For each condition we test for normality with the Shapiro-Wilk Normality Test. All cases except one (growth rates for isolates with no CTX-M-15 and TEM-1 in presence of cefepime) indicate normality (with p < 0.01).

	CTX-M-15 (+) and TEM-1 (+)	CTX-M-15 (+) and TEM-1 (-)	CTX-M-15 (-) and TEM-1 (+)	CTX-M-15 (-) and TEM-1 (-)
Ceftazidime	0.5647	0.5349	0.6467	0.07002
Ceftriaxone	0.8200	0.1587	0.1082	0.3209
Cefepime	0.2844	0.1986	0.0017	0.1255

## Results and discussion

The growth rates of these isolates were found using the optical density (OD) measurements of their growth in MH medium with a single concentration of the antibiotics CAZ, CRO, FEP, and AMP. We began by determining how well these growth rates corresponded to clinical determinations of susceptibility (Table 3). For simplicity, and because of sample size, we included only resistant and susceptible classifications. All isolates were either resistant or susceptible to FEP and CRO, and all isolates were found to be resistant to AMP. Three of the isolates displayed intermediate susceptibility to CAZ and were excluded from this analysis.

**Table 3:** Susceptibility to ceftazidime (CAZ), ceftriaxone (CRO), cefepime (FEP) and ampicillin (AMP). Clinical susceptibility classifications of isolates in the presence of four antibacterial agents. *Three intermediate samples excluded.

	CAZ*	CRO	FEP	AMP
Resistant	30	35	31	47
Susceptible	14	12	16	0

### Isolate growth rates and clinical antibiotic susceptibility tests

In the case of all three cephalosporin antibiotics (CAZ, CRO, and FEP), growth rates for isolates clinically determined to be resistant were higher than growth rates of susceptible isolates (Table 4 and Fig 1, p<2e-3). The entries marked with an asterisk in Table 4 indicate that one (or both) of the growth rate groups under analysis did not pass a test for normality (Table 1).

**Table 4:** Average growth rates (min^-1^) for all conditions. Mean isolate growth rates and 99% confidence intervals (CI) for the difference in mean growth rates based on a t-statistic.

	Mean Growth Rates		99% CI
	μ_1_	μ_2_	μ_1_-μ_2_
	Resistant	Susceptible	
CAZ	0.00880	0.00278	(0.00397, 0.00808)
CRO	0.01330	0.00361	(0.00674, 0.0126)
FEP*	0.01162	0.00555	(0.00145, 0.0107)
	CTX-M-15 (+)	CTX-M-15 (-)	
CAZ	0.00919	0.00325	(0.00403, 0.00787)
CRO*	0.01341	0.00666	(0.00304, 0.0104)
FEP	0.01262	0.00462	(0.00439, 0.0116)
	TEM-1 (+)	TEM-1 (-)	
CAZ	0.00517	0.00859	(-0.00629, -0.000551)
CRO	0.00773	0.01379	(-0.00971, -0.00240)
FEP*	0.00701	0.01199	(-0.00906, -0.000896)
	CTX-M-15 (+) and TEM-1 (+)	CTX-M-15 (+) and TEM-1 (-)	
CAZ	0.00808	0.00978	(-0.00559, 0.00220)
CRO	0.01148	0.01442	(-0.00847, 0.00257)
FEP*	0.01162	0.01314	(-0.00672, 0.00366)
	CTX-M-15 (+) and TEM-1 (-)	CTX-M-15 (-) and TEM-1 (+)	
CAZ	0.00978	0.00293	(0.00453, 0.00917)
CRO	0.01442	0.00485	(0.00593, 0.0132)
FEP*	0.01314	0.00347	(0.00621, 0.0131)
	CTX-M-15 (+) and TEM-1 (+)	CTX-M-15 (-) and TEM-1 (+)	
CAZ	0.00808	0.00293	(0.00142, 0.00890)
CRO	0.01148	0.00485	(0.000812, 0.0124)
FEP*	0.01162	0.00347	(0.00268, 0.0136)

The cephalosporin CAZ had the greatest inhibitory effect on the growth rates of both resistant and susceptible isolates (Fig 1). We believe this is due to the high frequency of the CTX-M-15 gene in our isolate population. The immediate ancestor of this gene, CTX-M-3 has innate activity against ceftriaxone and cefepime, and through mutation, CTX-M-15 more recently evolved activity against ceftazidime (see also Fig 3) [26]. All isolates were clinically determined to be resistant to ampicillin, and so we could not compare susceptible and resistant isolate growth rates.

### Resistance genes and isolate growth rates

**Fig 2:**
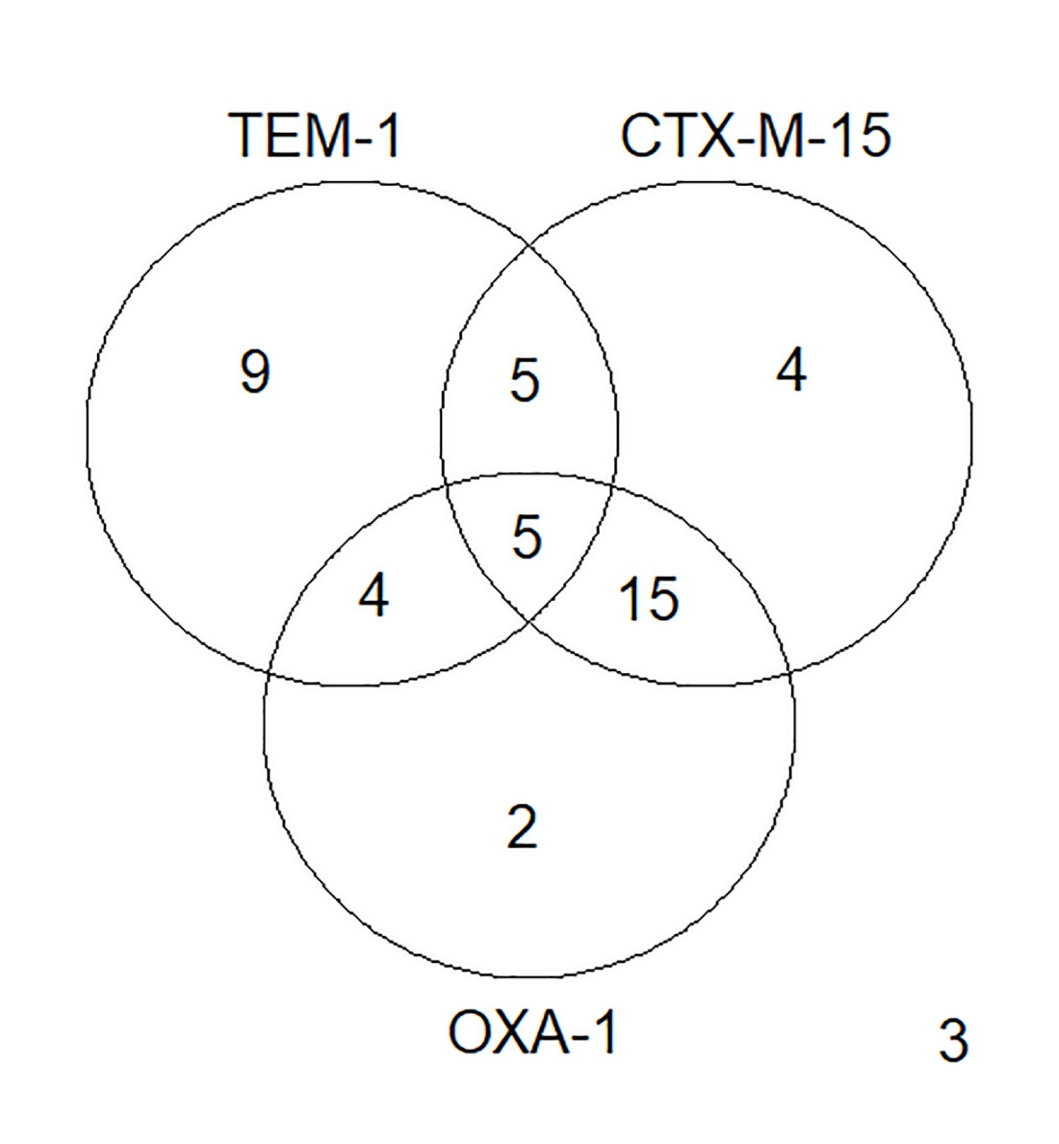
Resistance gene combinations in our 47-isolate population.

After confirming that growth rates are associated with the broad clinical categorization of resistance to cephalosporins, we wanted to analyze the effect of individual resistance genes on isolate growth rates. We hypothesize that isolates with genes known to confer resistance to cephalosporins would have higher growth rates than isolates without those resistance genes. We first identified the β-lactamases in our isolates (Table 5). Only three β-lactamases occurred at high frequency (more than 20 isolates) in our data set: CTX-M-15, TEM-1 and OXA-1 (Table 5, Fig 2). Although we observed four other β-lactamases: TEM-19 (1 isolate), CTX-M-14 (4 isolates), CTX-M-27 (3 isolates), CTX-M-65 (2 isolates), we focused on CTX-M-15, TEM-1 and OXA-1 because of their frequency.

**Table 5:** Presence of CTX-M-15, TEM-1, and OXA-1 resistance genes. The frequency of resistance genes CTX-M-15, TEM-1, and OXA-1 in our 47 isolates.

	CTX-M-15	TEM-1	OXA-1
Present (+)	29	23	26
Absent (-)	18	24	21

### Observed effect of CTX-M-15 on isolate growth rates

**Fig 3:**
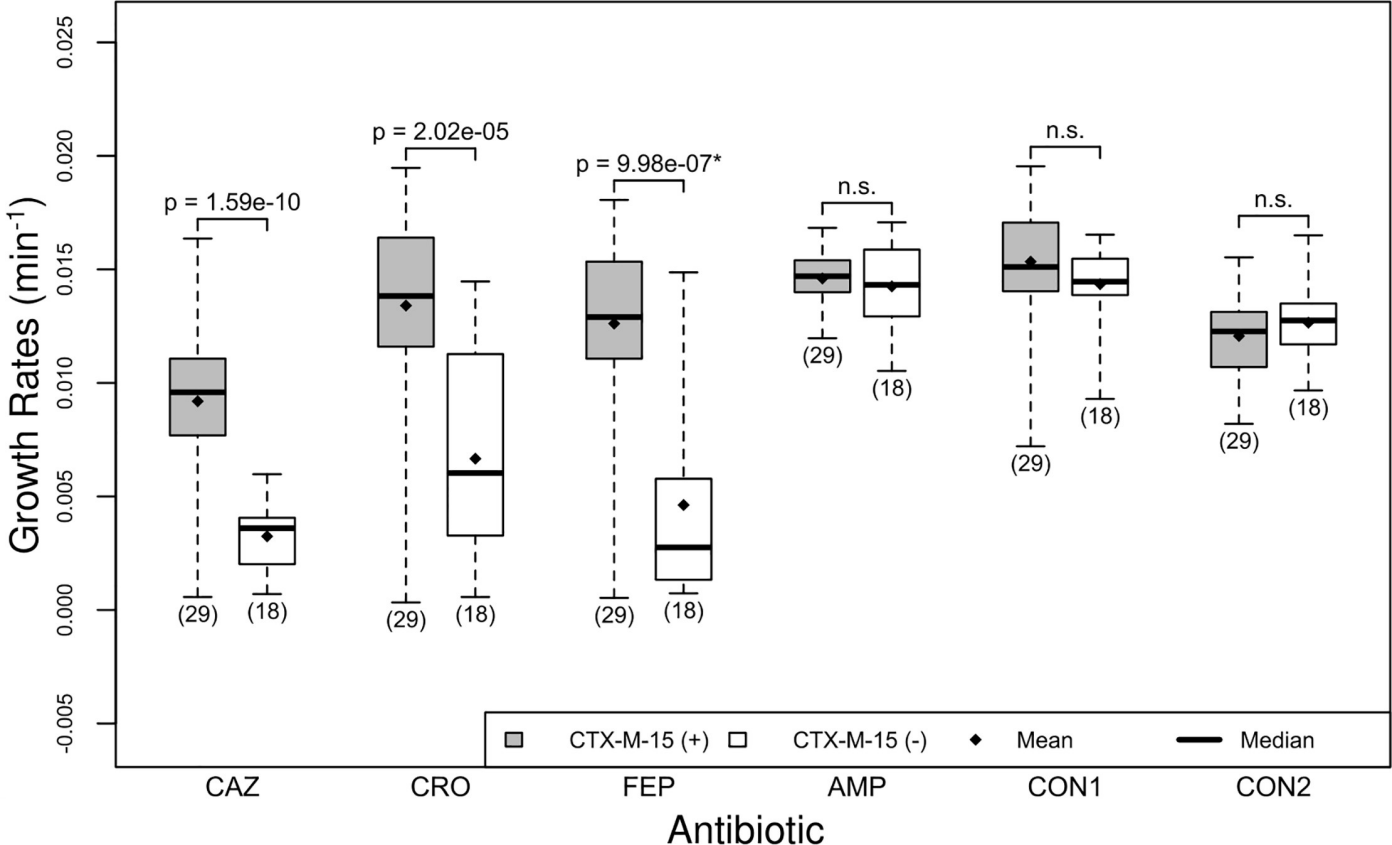
Isolates with CTX-M-15 exhibit higher growth rates in the presence of cephalosporins. There are two boxplots per antibiotic, the first (gray) shows the distribution of growth rates for isolates that have the CTX-M-15 gene, labeled CTX-M-15 (+) and the second (white) shows the distribution of growth rates for isolates that do not have the CTX-M-15 gene, labeled CTX-M-15 (-). The n.s. indicates the difference in growth rates is not significant. Abbreviations for controls: CON1, the control growth rates for the experiment CAZ, CRO and FEP; CON2, the control growth rates for the experiment AMP. See Fig 1 for interpretation of boxplots.

We found that isolates with the CTX-M-15 gene had a higher growth rate in the presence of all three cephalosporin antibiotics than isolates that did not have the resistance marker (Table 4, Fig 3, p<2.05e-5). We did not observe this difference in either of the two controls (i.e., isolates grown with no antibiotic) or isolates grown in the presence of ampicillin.

### Observed effect of TEM-1 on isolate growth rates

**Fig 4:**
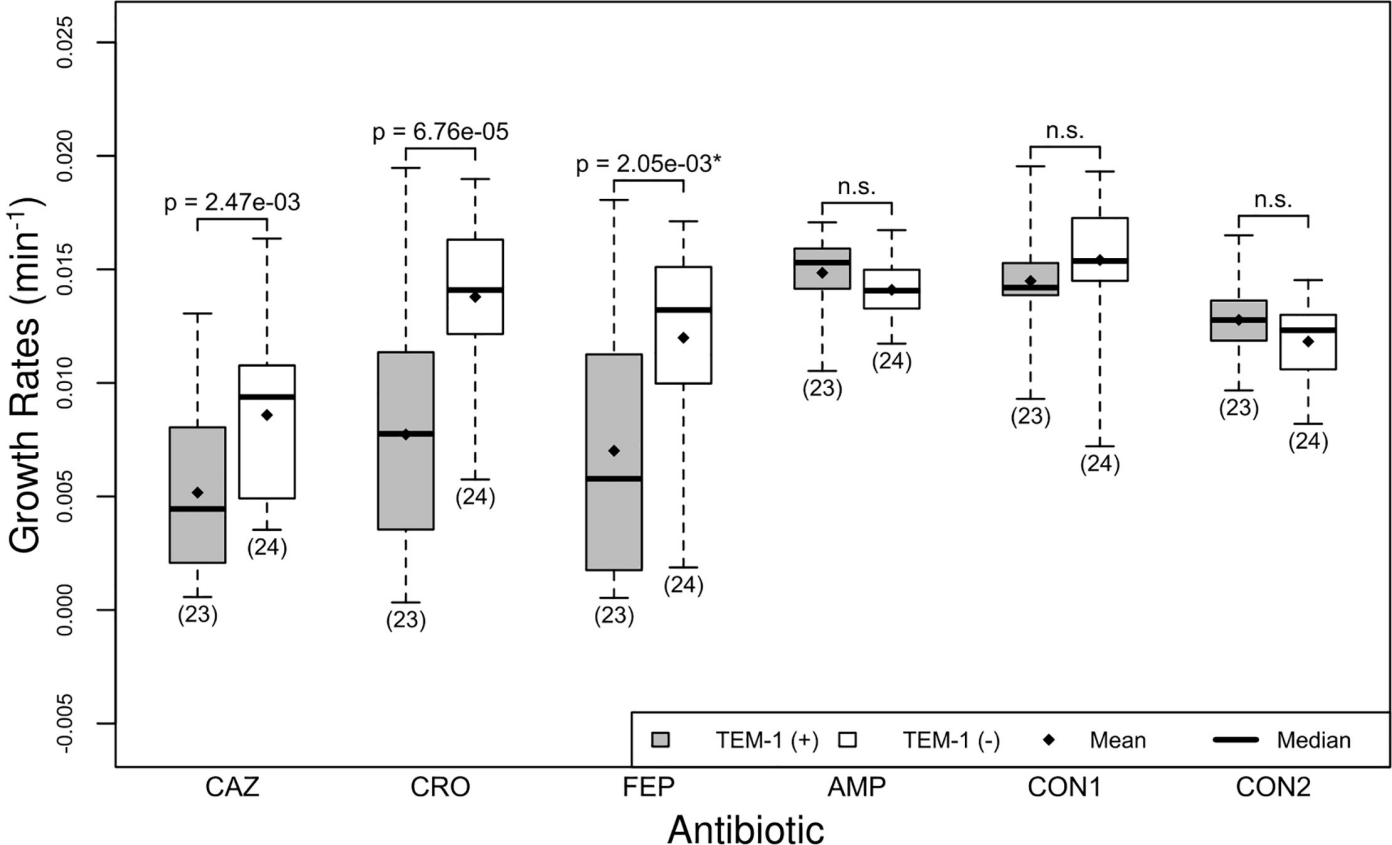
Isolates with TEM-1 exhibit lower growth rates in the presence of cephalosporins. There are two boxplots per antibiotic, the first (gray) shows the distribution of growth rates for isolates that have the TEM-1 gene, labeled TEM-1 gene (+), and the second (white) shows the distribution of growth rates for isolates that do not have the TEM-1 gene, labeled TEM-1 (-). The n.s. indicates the difference in growth rates is not significant. See Fig 1 for interpretation of boxplots.

When comparing the growth rates of isolates with the TEM-1 gene and those without the gene, we observed that isolates without the TEM-1 gene had a higher growth rate than those that had the marker (Table 4, Fig 4, p<2.5e-3). This difference in growth rates was not observed in the ampicillin experiments or the controls.

There are multiple potential causes for this effect that we have not yet explored which may not directly result from TEM-1 expression. As CTX-M is a powerful cephalosporinase, the fact that our isolates without TEM-1 were likely to have CTX-M-15 may be the primary reason for these differences (Fig 2). However, the difference in frequency has been observed but not addressed as CTX-M genes have replaced TEM genes in bacterial populations [16], [17], [18]. This unexplained shift in populations suggests that something more may be at work. Another possibility is that the two genes result in negative sign epistasis when they co-occur. Since the TEM-1 enzyme appears to hydrolyze cephalosporins more slowly than CTX-Ms, its presence could interfere with CTX-M hydrolysis of cephalosporins and decrease their overall efficiency. While we do not fully understand the biochemical basis for such a fitness cost, it does provide a mechanistic reason for the observation that CTX-M-15 and OXA-1 are replacing TEM-1 in bacterial populations [16].

### Observed effect of OXA-1 on isolate growth rates

**Fig 5:**
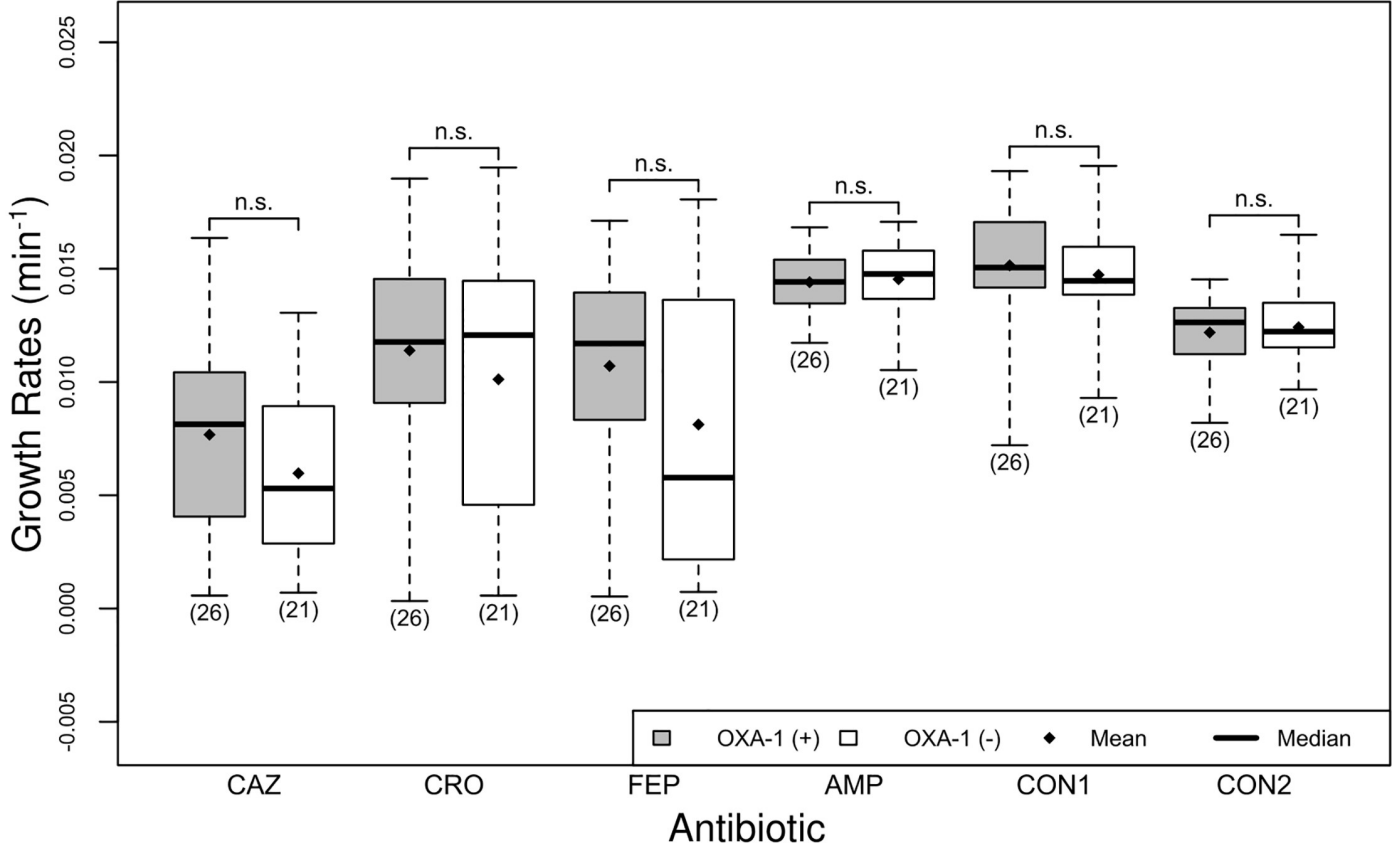
The presence of OXA-1 does not affect isolate growth rates. There are two boxplots per antibiotic, the first (gray) shows the distribution of growth rates for isolates that have the OXA-1 gene, labeled OXA-1 gene (+), and the second (white) shows the distribution of growth rates for isolates that do not have the OXA-1 gene, labeled OXA-1 (-). See Fig 1 for interpretation of boxplots.

We did not observe a difference in growth rates based on the presence or absence of the OXA-1 gene (Fig 5). This may be due to the high frequency of the co-occurrence of CTX-M-15 and OXA-1 (Fig 2). Also, OXA-1 is penicillinase whose emergence was thought to result from the clinical introduction of oxacillin and methicillin. The different antibiotic specificities of OXA-1 and CTX-M-15 may confer an advantage when bacteria expressing both are exposed to a wide variety of antibiotics [27].

### Separate and combined effects of CTX-M-15 and TEM-1 on isolate growth rates

**Fig 6:**
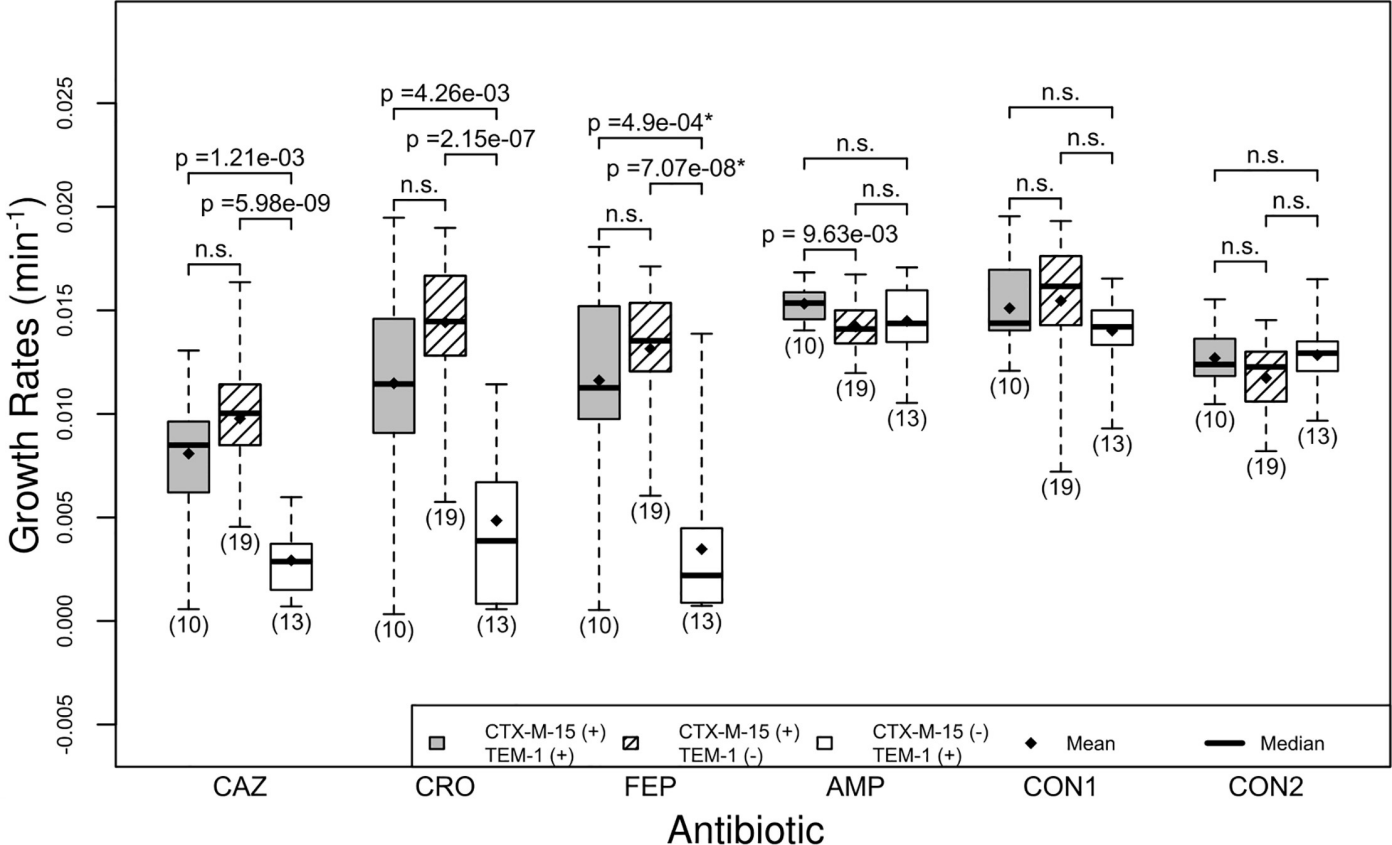
Isolates with CTX-M-15 exhibit higher growth rates irrespective of TEM-1 presence. There are three boxplots per antibiotic, the first boxplot (gray) shows the distribution of growth rates for isolates that have the CTX-M-15 gene and the TEM-1 gene (labeled CTX-M-15 (+) and TEM-1 (+)), the second boxplot (hash marked) shows the distribution of growth rates for isolates that have the CTX-M-15 gene but not the TEM-1 gene (labeled CTX-M-15 (+) and TEM-1 (-)), and the third boxplot (white) shows the distribution of growth rates for isolates that have the TEM-1 gene but not the CTX-M-15 gene (labeled CTX-M-15 (-) and TEM-1 (+)). See Fig 1 for interpretation of boxplots.

Based on our previous observations about CTX-M-15 and TEM-1 in our isolates, we considered their combined effects. We found that the presence of TEM-1 is not significantly associated with changes in growth rates for isolates that carry CTX-M-15 even though there is a slight increase in mean growth rate for isolates lacking TEM-1 (Table 4, Fig 6). It is possible that this result may become significant in future studies as the sample size increases since there is a tendency for isolates without TEM-1 to have a faster growth rate than those that have it (Figs 4 and 6). Additionally, in the presence of ampicillin, isolates carrying TEM-1 had a statistically significant faster growth rate (Table 4, Fig 6, p<9.64e-3). This result is expected because TEM-1 is an efficient penicillinase whereas CTX-M-15 is primarily a cephalosporinase.

## Conclusion

The continued evolution of bacterial resistance to clinically useful antibiotics is a major world health crisis. The ability of bacteria to evolve and transfer resistance genes rapidly throughout a bacterial population presents an ongoing challenge for healthcare providers and will require the development of more effective treatment plans. Coping with this challenging problem requires not only effort of clinicians, but the expertise of researchers in evolutionary biology, statistics and mathematics. Methods in these latter fields require sensitive techniques for quantifying the fitness of a bacterial isolate to an antibiotic. We propose that bacterial growth rates have the potential to be a useful technique to the study of antibiotic resistance in these fields because growth rates can provide more information about the fitness effects of resistance genes than current clinical classifications of resistance.

In this study, we used *E*. *coli* isolates from Dignity Health, Mercy Medical Center to measure bacterial growth rates in the presence of different antibiotics. In particular, we determined growth rates for each of 47 bacterial isolates in the presence of three cephalosporins and one penicillin. Through growth rate assays, we were able to quantify an isolate’s fitness in the presence of antibiotics at single concentrations and found these results to be highly consistent with the information provided by clinical antibiotic susceptibility testing. Additionally, the sensitivity of growth rates enabled us to observe the effects that resistance genes had on isolate growth rates, both individually and in various combinations. We also confirmed that CTX-M-15 is an excellent cephalosporinase.

Overall, we found that growth rates have greater sensitivity for assaying interactions between resistance genes and may be useful in helping to develop predictive models for assessing antibiotic susceptibility based on presence or absence of resistance genes. While growth rates are not a good candidate for susceptibility testing, their added sensitivity may help reveal the interactions between resistance genes and may facilitate improvements in the reliability of molecular diagnostic methods. Our findings suggest that there may be inhibitory interactions between resistance genes, and we intend to explore the relationships in future studies with isogenic strains.

## Supporting information

S1 Fig Boxplot of *E*.*coli* isolates growth rates in the presence of ceftazidime at a concentration of 64 μg/mL. The boundaries on the boxes indicate the 25^th^ (Q1) and the 75^th^ (Q3) percentiles (quartiles), the line in the box represents the median, and the whiskers indicate the minimum (below) and maximum (above) growth rate. There are 214 boxplots, one box-plot per isolate from six technical replicates. This figure shows that at a concentration of 64 μg/mL, we observe phenotypic differences between isolate growth rates.
(TIF)

S2 Fig Boxplot of *E*.*coli* isolates growth rates in the presence of ceftriaxone at a concentration of 64 μg/mL. The boundaries on the boxes indicate the 25^th^ (Q1) and the 75^th^ (Q3) percentiles (quartiles), the line in the box represents the median, and the whiskers indicate the minimum (below) and maximum (above) growth rate. There are 214 boxplots, one box-plot per isolate from six technical replicates. This figure shows that at a concentration of 64 μg/mL, we observe phenotypic differences between isolate growth rates.
(TIF)

S3 Fig Boxplot of *E*.*coli* isolates growth rates in the presence of cefepime at a concentration of 64 μg/mL. The boundaries on the boxes indicate the 25^th^ (Q1) and the 75^th^ (Q3) percentiles (quartiles), the line in the box represents the median, and the whiskers indicate the minimum (below) and maximum (above) growth rate. There are 214 boxplots, one box-plot per isolate from six technical replicates. This figure shows that at a concentration of 64 μg/mL, we observe phenotypic differences between isolate growth rates.
(TIF)

S4 Fig Optical density (OD) measurements of *E*.*coli* isolates in the presence of ceftriaxone at a concentration of 64 μg/mL and control over a period of 22 hours. OD measurements were made at 600nm every 20 minutes. The circles represent OD measurements for isolate99; the squares represent the OD measurements for isolate155; the triangles represent the OD measurements for isolate109; and the diamonds represent the OD measurements for isolate105.
(TIF)
