# Supplementary figures and images for: Growth rate assays reveal fitness consequences of β-lactamases
Source: PLoS One. 2020 Jan 31;15(1):e0228240. doi: 10.1371/journal.pone.0228240 (PMC6993977; doi:10.1371/journal.pone.0228240)

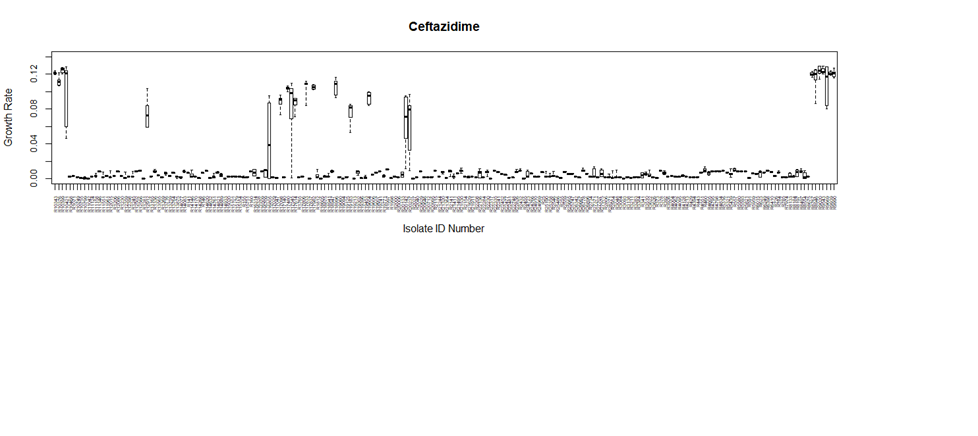

Supplement: S1 Fig — The boundaries on the boxes indicate the 25th (Q1) and the 75th (Q3) percentiles (quartiles), the line in the box represents the median, and the whiskers indicate the minimum (below) and maximum (above) growth rate. There are 214 boxplots, one box-plot per isolate from six technical replicates. This figure shows that at a concentration of 64 μg/mL, we observe phenotypic differences between isolate growth rates. (TIF) [file pone.0228240.s001.tif]

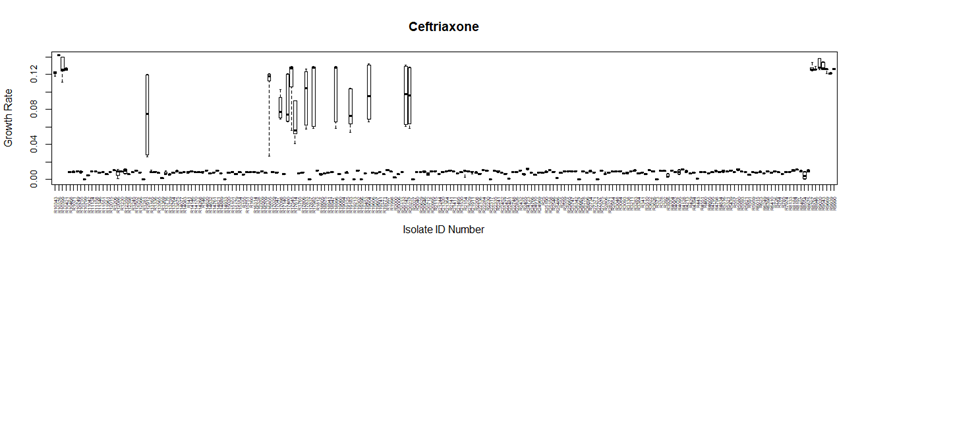

Supplement: S2 Fig — The boundaries on the boxes indicate the 25th (Q1) and the 75th (Q3) percentiles (quartiles), the line in the box represents the median, and the whiskers indicate the minimum (below) and maximum (above) growth rate. There are 214 boxplots, one box-plot per isolate from six technical replicates. This figure shows that at a concentration of 64 μg/mL, we observe phenotypic differences between isolate growth rates. (TIF) [file pone.0228240.s002.tif]

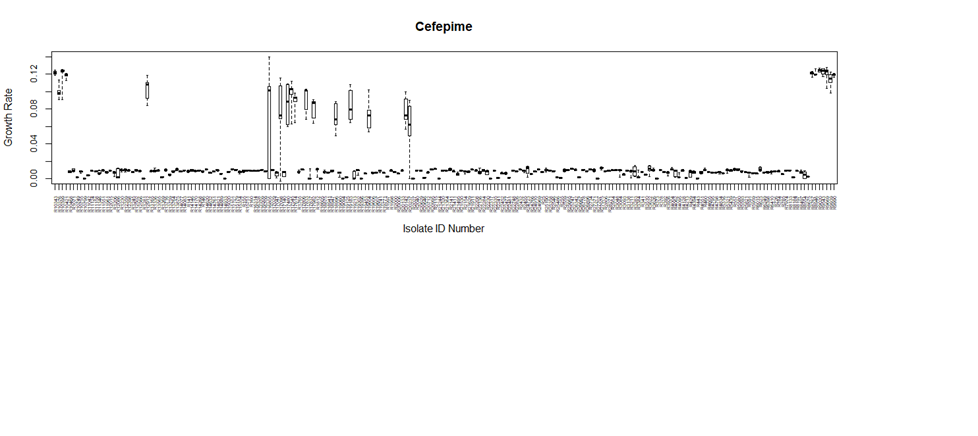

Supplement: S3 Fig — The boundaries on the boxes indicate the 25th (Q1) and the 75th (Q3) percentiles (quartiles), the line in the box represents the median, and the whiskers indicate the minimum (below) and maximum (above) growth rate. There are 214 boxplots, one box-plot per isolate from six technical replicates. This figure shows that at a concentration of 64 μg/mL, we observe phenotypic differences between isolate growth rates. (TIF) [file pone.0228240.s003.tif]

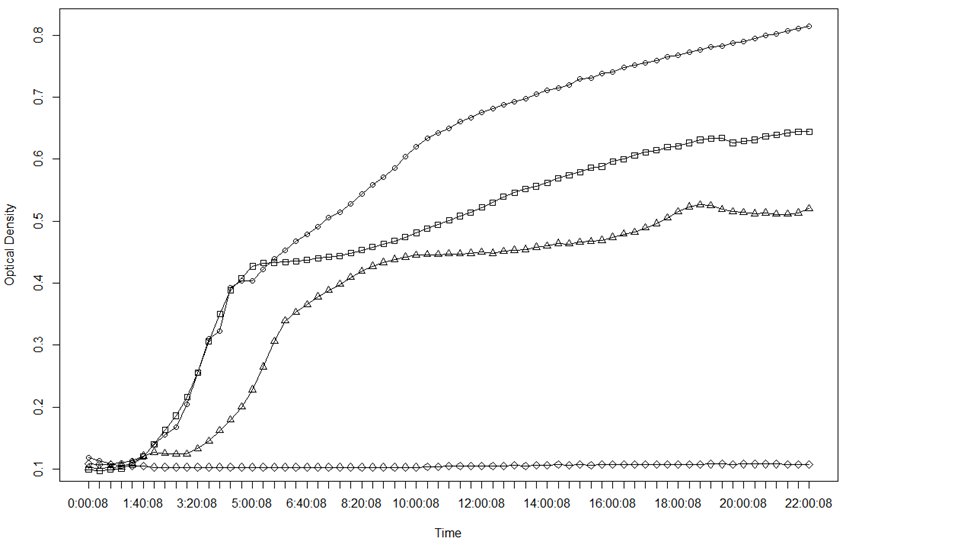

Supplement: S4 Fig — OD measurements were made at 600nm every 20 minutes. The circles represent OD measurements for isolate99; the squares represent the OD measurements for isolate155; the triangles represent the OD measurements for isolate109; and the diamonds represent the OD measurements for isolate105. (TIF) [file pone.0228240.s004.tif]
